# Cerebral Vasculitis Revealing Systemic Sarcoidosis: A Case Report and Review of the Literature

**DOI:** 10.7759/cureus.36968

**Published:** 2023-03-31

**Authors:** Christ Labretesche Gracia Gakosso, Slioui Badr, Yousra Zouine, Nabil Hammoune, Abdelilah Mouhsine

**Affiliations:** 1 Department of Radiology, Avicenne Military Hospital, Faculty of Medicine and Pharmacy, Cadi Ayyad University, Marrakech, MAR

**Keywords:** sarcoidosis, ischemia, hemorrhage, imaging, cerebral vasculitis, neurosarcoidosis

## Abstract

Vasculitis in neurosarcoidosis is rare, with only a few cases reported in the literature. We report the clinical observation of a 51-year-old patient with no previous medical history, who was admitted to the emergency department due to sudden onset confusion, fever, sweating, weakness, and headaches. The first brain scan was normal, but a further biological exam with a lumbar puncture revealed lymphocytic meningitis. A complementary cerebral MRI revealed abnormalities in the white matter signal, suggestive of multiple sclerosis, with petechial hemorrhagic foci associated with leptomeningeal involvement and cerebral vasculitis. Thoraco-abdomino-pelvic computed tomography revealed hilar and mediastinal lymphadenopathy, as well as lymph nodes in the lower cervical region. A biopsy of the lymph nodes confirmed the presence of non-caseating granulomatous inflammation consistent with sarcoidosis. High-dose corticosteroid therapy was initiated with good clinical outcomes. Cerebral vasculitis in neurosarcoidosis is rare but can lead to neurological complications requiring long-term multidisciplinary management.

## Introduction

Sarcoidosis is a multisystemic, inflammatory, and idiopathic granulomatous disease that typically affects the lungs, uvea, lymph nodes, and skin [1,2]. Clinical manifestations include general signs such as night sweats, fatigue, and weight loss, as well as signs specific to the region or organ affected by sarcoidosis [3]. The occurrence of cerebral vasculitis in neurosarcoidosis is a rare and unusual manifestation of the disease [2-4]. We report a case of cerebral vasculitis revealing neurosarcoidosis.

## Case presentation

**Figure 1:**
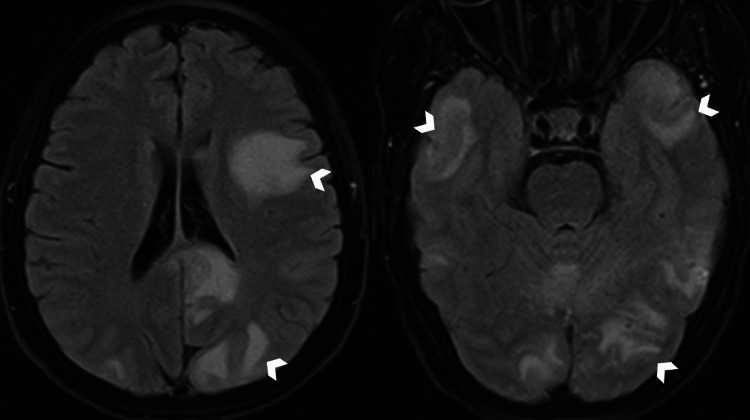
Axial slices in the FLAIR sequence showing hyperintensities in bilateral subcortical and periventricular hemispheric white matter, involving U-fibers and the splenium of the corpus callosum. FLAIR: fluid-attenuated inversion recovery.

**Figure 2:**
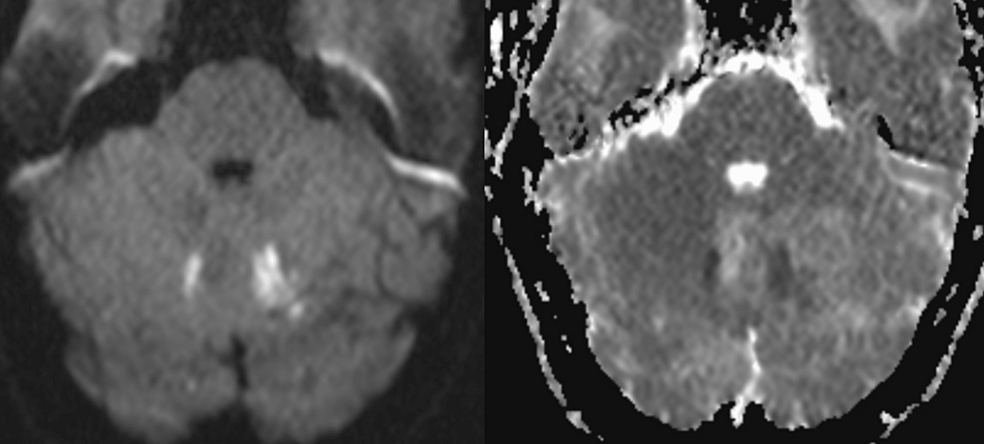
Diffusion-weighted sequence with ADC mapping showing bilateral cerebellar hyperintensity (left image) with low ADC (right image). ADC: apparent diffusion coefficient.

**Figure 3:**
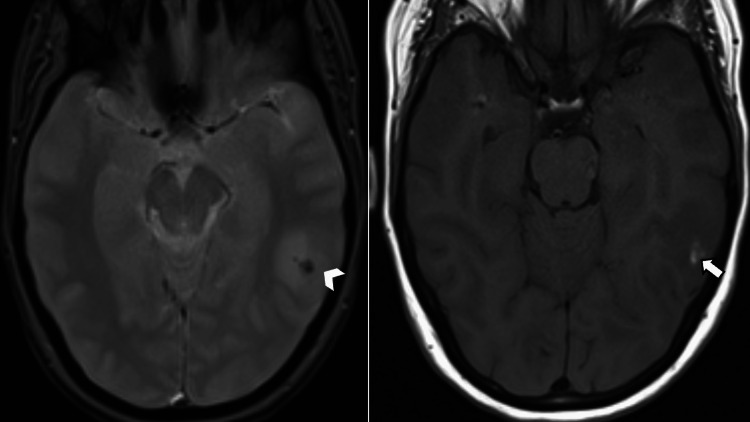
Gradient echo T2-weighted and T1-weighted sequences showing a left parietal hemorrhagic spot (arrowhead), which appears as a hyperintense signal on the T1 sequence (arrow).

**Figure 4:**
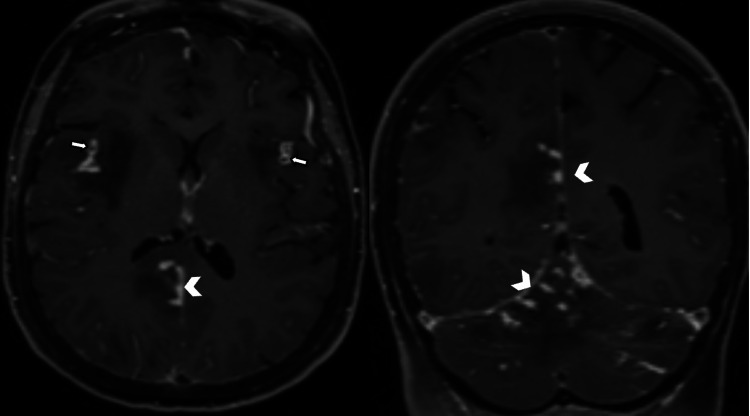
Axial and coronal T1 sequences after gadolinium injection and fat suppression showing contrast enhancement of vessel walls (arrows) associated with micronodular thickening of the meningeal layers (arrowheads).

A 51-year-old woman was admitted to the emergency department for sudden-onset mental confusion in the context of fever, sweating, fatigue, and moderate-intensity pulsatile headaches without visual disturbances, which were only relieved after taking painkillers. On clinical examination, the patient was hemodynamically stable with a blood pressure of 115/80 mmHg, a pulse rate of 70 beats/minute, and a respiratory rate of 18 cycles/minute. Neurological examination did not reveal any motor deficits but showed painful neck stiffness without other associated neurological symptoms. Examination of the thoracic and abdominal regions was unremarkable. Initial paraclinical investigations included laboratory tests showing elevated C-reactive protein (CRP) and erythrocyte sedimentation rate. The non-contrast brain CT scan was normal, and a cerebrospinal fluid (CSF) examination was performed, revealing a normal pressure of 180 mmHg and lymphocytic meningitis with 47 cells and hyperproteinemia. Further imaging with MRI was requested by the neurologist, which revealed multiple abnormalities in the signal of bilateral subcortical hemispheric white matter with U-fiber involvement showing hyperintensity on fluid-attenuated inversion recovery (FLAIR) sequence (Figure 1), some of which were restricted in diffusion-weighted imaging (DWI) and dark on apparent diffusion coefficient (ADC) (Figure 2). Additionally, there were petechial hemorrhagic foci with hypointensity on T2* gradient-echo magnetic susceptibility sequence and hyperintensity on T1 (Figure 3), along with a micronodular meningeal thickening and intracranial vascular wall enhancement on T1 fat-suppressed sequence with gadolinium injection (Figure 4). As part of the etiological investigation, a thoraco-abdomino-pelvic CT scan was performed, revealing multiple hilar and lower cervical mediastinal lymphadenopathies at the thoracic level. An ultrasound-guided biopsy of the cervical lymphadenopathy showed giant cell epithelioid granuloma without caseous necrosis suggestive of sarcoidosis. The patient was started on corticosteroid therapy (five doses of 1 gram of intravenous methylprednisolone) with the improvement of the headache and disappearance of cervical stiffness, as well as normalization of the inflammatory biological tests, including CRP.

## Discussion

Central nervous system involvement in the context of sarcoidosis affects 5% to 10% of all patients according to the literature [1,2]. Due to its unusual occurrence, sarcoidosis rarely causes vasculitis in the cerebral nervous system [3]. All parts of the nervous system and its coverings can be affected in neurosarcoidosis, including the cerebral parenchyma, nerve roots, meninges, dura mater, and adjacent bone structures [3,4]. Less commonly, sarcoid granulomas can cause perivascular inflammation and infiltrate the walls of intracranial blood vessels, leading to cerebrovascular events such as infarction, venous thrombosis, hemorrhages, and intracranial vasculitis [4-6]. In addition, it should be noted that certain radiological features of neurological involvement are strongly suggestive of the diagnosis, notably micronodular thickening of the meninges and hypothalamic-pituitary involvement. The neurological manifestation depends on the site and extent of central nervous system involvement [7]. More often, cranial nerve neuropathy, meningitis, hydrocephalus, and encephalitis, and, less commonly, an ischemic or hemorrhagic stroke and intra-parenchymal inflammatory pseudotumors are found [5-7]. From a radiological point of view, MRI is the technique of choice for exploring cerebral abnormalities in sarcoidosis in the initial phase as well as during follow-up [3]. As reported in the literature, a cerebral MRI was performed in our patient following a normal cerebral CT scan. In MRI, neurosarcoidosis more often affects the base of the skull and the cranial nerves [1-4]. Nervous system involvement of the facial nerve (VII) and the optic nerve and its chiasm is revealed by a swollen appearance with iso-signal T1, hyper-signal T2, and FLAIR, and enhanced after gadolinium injection [2,3]. In cases of parenchymal involvement, lesions of the white matter are found, similar to those in multiple sclerosis with U-fiber involvement and hyper-signal FLAIR and T2 not enhanced after gadolinium injection [3-5]. In the case of meningeal involvement, there is diffuse or focal micronodular thickening, predominantly at the base of the skull, supra-sellar, and fronto-basal regions, hyper-signal T2 and enhanced after gadolinium injection, forming in some cases true extra-axial pseudomasses by coalescence of sarcoid granulomas [2-4,7]. They can extend by extension toward the hypothalamo-hypophyseal axis, appearing as iso signal T1, iso, or hyposignal T2 enhanced after gadolinium injection [3,4]. More rarely, and as reported in our case, vasculitis due to cerebral sarcoidosis can occur, illustrated on MRI either by thickening and contrast uptake of vascular walls after gadolinium injection or in some cases by intracranial hemorrhages manifesting as hyposignals (microbleeds) on magnetic susceptibility sequences (gradient echo T2 or susceptibility weighted imaging) and venous or arterial thromboses of the stroke type [5,6]. Therefore, the diagnosis comes from a bundle of arguments, including clinical, radiological, biological, and histological results. Other etiologies of central nervous system vascular disease include infectious, autoimmune, and other inflammatory diseases. These must be excluded by a well-conducted history, clinical examination, and appropriate investigations [4]. The treatment is mainly based on first-line corticosteroid therapy, sometimes in combination with immunosuppressants [7-9]. Our patient received corticosteroid therapy consisting of five doses of 1 gram of intravenous methylprednisolone with good clinical improvement resulting in the disappearance of meningeal stiffness, fever, and headaches, which is consistent with the literature.

## Conclusions

Cerebral vasculitis as the initial manifestation of neurosarcoidosis is rare, unusual, and carries a poor prognosis. Therefore, this clinical case teaches us that every physician should consider sarcoid arteritis in the etiological assessment of cerebrovascular disease. MRI, when accessible, is the examination of choice in exploring this vasculitis. Early initiation of appropriate treatment can prevent the development of serious complications and offer good clinical outcomes.
